# Responses of functional traits in cavity-nesting birds to logging in subtropical and temperate forests of the Americas

**DOI:** 10.1038/s41598-021-03756-0

**Published:** 2021-12-21

**Authors:** Alejandro A. Schaaf, Daniela Gomez, Ever Tallei, Constanza G. Vivanco, Román A. Ruggera

**Affiliations:** 1 Instituto de Ecorregiones Andinas (INECOA), Universidad Nacional de Jujuy – Centro Científico Tecnológico CONICET Salta-Jujuy, Av. Bolivia 1239, 4600 San Salvador de Jujuy, Jujuy, Argentina; 2 Biología de Aves, Instituto Argentino de Investigaciones de Las Zonas Áridas (IADIZA), Centro Científico Tecnológico CONICET – Mendoza, Av. Ruiz Leal s/n, Parque General San Martin, 5500 Mendoza, Argentina

**Keywords:** Ecology, Ecology

## Abstract

Logging causes changes in habitat structure, which can potentially lead to variations in taxonomic and functional richness of biodiversity. Studies on how functional traits in birds are affected by logging operations can provide an important element for the understanding of ecosystem processes. In this paper, we examined how logging in subtropical Andean forests influenced taxonomic and functional diversity of cavity-nesting birds. We used these results to compare how logging affected ecosystem functions in temperate and subtropical forests of the Americas. We used point-counts to examine the effects of logging on taxonomic and functional traits in avian communities (Functional Richness, Functional evenness, Functional Divergence, and Community-weighted mean). We found that logging changed bird richness and abundance, although it had no effect on the functional response to the measured traits. The comparison of our results with those of temperate forests of Canada and Chile reveals differences in the functional richness of birds in these habitats, with a lower impact of logging on functional traits. We highlight the importance of including functional traits in the analyses, since the reduction in the species richness and abundance may not be translated into functional changes within the ecosystem.

## Introduction

Severe anthropogenic disturbances, such as fragmentation, habitat loss, and selective logging are major threats to worldwide biodiversity^1,2^. In particular, the loss of tropical and subtropical native forests due to selective logging can result in negative changes to vertebrates’ community composition^3,4^ and, particularly that of birds. When logging is conducted, the extraction of different tree species changes the availability of food and nesting resources^5,6^. In general, logging leads to an increase in the abundance of widely distributed bird species with low resource use specificity (e.g. feeding or nesting)^7–9^. Other bird species, however, may respond negatively to these changes, with a significant decrease in the composition, number and abundance of birds^9–12^.

Approximately 18% of all the bird species in the world use tree cavities as nesting sites^13^, resulting in trees being a key resource in forest ecosystems for the reproduction of birds^14–16^. Some species excavate their own cavities (e.g. woodpeckers and trogons), whereas non-excavator birds (e.g. owls, parrots) use cavities excavated by woodpeckers or cavities formed through decaying wood^13,15^. Current evidence in tropical and subtropical forests indicates that cavity-nesting birds are one of the most sensitive groups to forest exploitation^9,17–20^. Logging may remove essential trees for reproduction of cavity-nesting birds, since certain species use trees with specific features (e.g. size, cavity depth, tree species)^14,21^. For example, large secondary cavity-nesting bird species (such as ducks, parrots and toucans) depend on trees with an appropriate entrance size and depth for nesting, which can be scarce or absent in logged forests^14,22,23^. For woodpeckers, habitat alteration can lead to difficulties in the use of substrates suitable for excavation^24–26^ and it can reduce the abundance of these birds^26,27^. Many of the species affected by logging play an important role as pest controllers (e.g. owls and raptors) or seed dispersers (e.g. toucans); therefore, the diversity and abundance of these species are crucial to the persistence of the ecosystem services in the forest^8,17,28,29^.

Traditionally, taxonomic indices analyzed changes in community diversity^30,31^. Nowadays these indices are complemented with functional diversity indices which describe how different species traits respond to ecosystem changes^32,33^. For birds, these indices have often been based on morphological (e.g. body mass) and ecological traits (e.g. trophic guilds, seed dispersal, pest control)^29,32^. For instance, species richness may increase or remain unchanged in response to environmental disturbance, whereas functional diversity can be sensitive to these changes^31,33,34^. This may occur because, in disturbed environments, there may be a replacement or decline of habitat-specialist species, with a consequent increase of generalist species^35–37^. Thus, measuring functional traits is important and necessary to understand the effect of environmental perturbation^38,39^.

Regarding cavity-nesting birds, preliminary work focusing on the effects of logging on species richness and functional diversity of bird species occurred in temperate forests of North (Canada) and South (Chile) America^29^. In this southern temperate forest, logging reduced species richness and functional diversity parameters. On the other hand, logging did not affect species richness but reduced functional parameters in the northern temperate forest^29^. This shows that although some species can persist in these habitats, their ecological functions may be severely affected^29^. Meanwhile, in subtropical forests of South America, specifically in Argentina, studies were conducted on the effect of logging on functional traits in cavity trees for secondary cavity-nesting birds^40^, but not on the effects of functional traits in cavity-nesting bird species. The main results of this study showed a negative effect in richness and functional parameters of cavity trees as a result of logging^40^. Therefore, the importance of carrying out studies that analyze and compare the effect of forest exploitation on taxonomic and functional diversity is crucial^33,41,42^, especially on cavity-nesting birds in temperate and subtropical forests in the Americas.

In this study, we assess taxonomic richness and functional diversity of cavity-nesting birds at both logged and unlogged Andean subtropical forests of northwestern Argentina. Based on the impact of logging on avian cavity nesters, and the results of prior studies in American temperate forests^29^, we expect lower taxonomic richness and functional diversity of these bird species in disturbed sites. Finally, we compare how the taxonomic and functional diversity of cavity-nesting birds respond to logging in temperate and subtropical forests of America.

## Methods

### Study area

This study took place in piedmont rainforest of northwestern Argentina, which belongs to the seasonally dry forests of South America located between 400 and 900 m a. s. l.^43^. The climate is highly seasonal with annual rainfall between 800 and 1000 mm, concentrated from November to February^44^. This forest is characterized by the presence of several dominant tree species: *Calycophylum multiflorum*, *Phyllostylon rhamnoides*, *Myroxylon peruiferum, Amburana cearensis,* and *Cedrela angustifolia.* Over the years, timber extraction on piedmont forests has been mainly focused on 10 tree species: *Cedrela balansae, A. cearensis, Anadenanthera colubrina, C. multiflorum, P. rhamnoides, Myracrodruon urundeuva, Tabebuia avellanedae, Myroxylon peruiferum, Cordia trichotoma,* and *Pterogyne nitens*^45^. The abundance of these species has been widely reduced in logged sites^46,47^: a volume of 4 m^3^/ha on overage is extracted, ranging from a minimum of 1 m^3^/ha to a maximum of 13 m^3^/ha. The largest volume of the harvested timber belongs to *M. peruiferum*, *A. colubrina*, *C. multiflorum*, *P. rhamnoides*, *C. balansae* and *Handroanthus impetiginosus*^48^. This logging has led to impoverished and simplified forests, with basal area values less than half of their potential^45^.

### Fieldwork: habitat structure and bird survey

**Figure 1:**
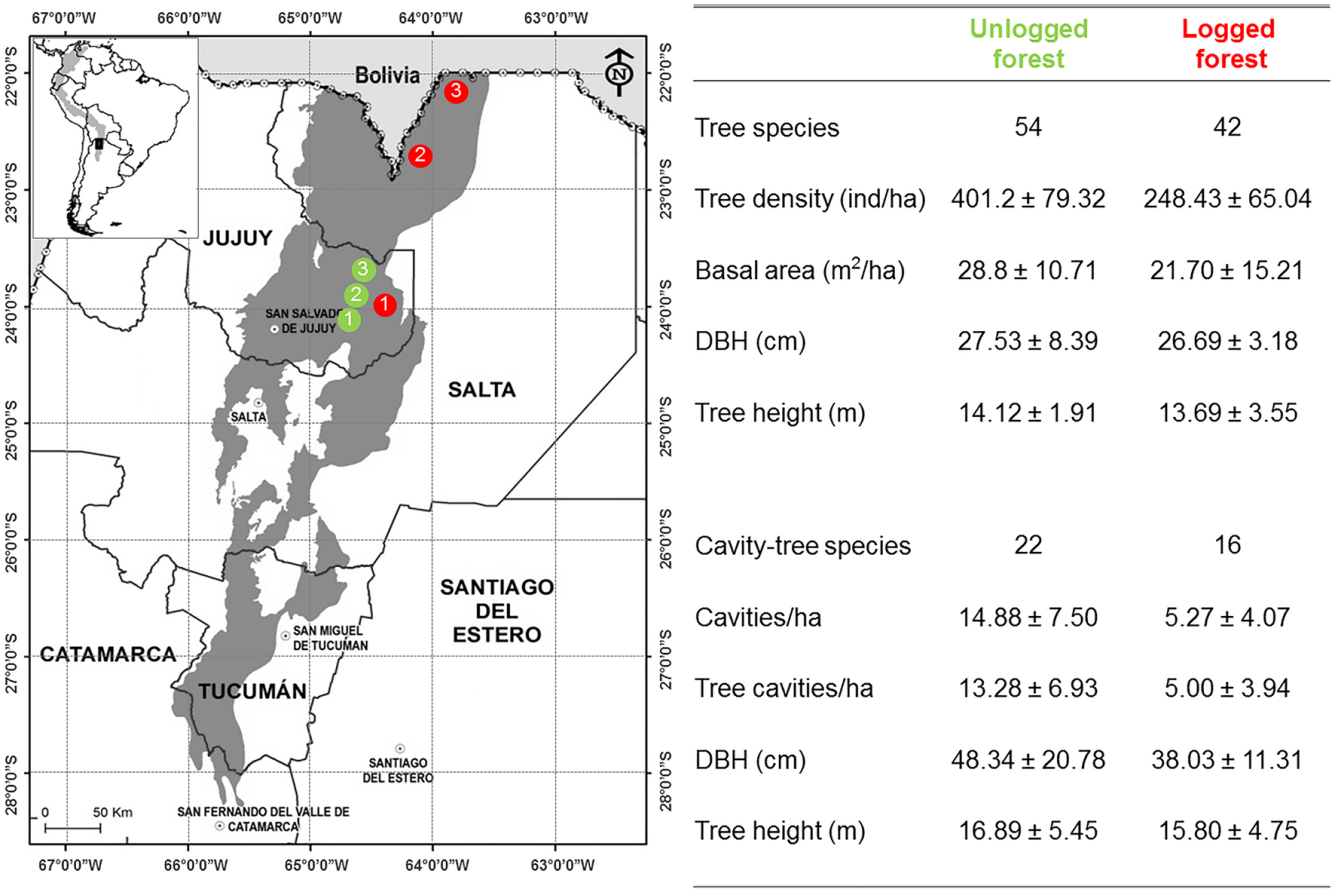
Location of the study sites in subtropical piedmont forests of northwestern Argentina: unlogged (green) and logged (red) forest sites are depicted on the map. Total tree species and habitat variables measured are also shown. This figure was produced using QGIS version 3.20.2 (https://www.qgis.org/).

Field sampling was carried out in six sites, three of which have not been logged for more than 45 years, and three with ongoing selective logging. We grouped treatments as ‘unlogged’ and ‘logged’ sites, respectively, for statistical analyses (Fig. 1). At each site, we delimited an area of 100 ha. Habitat characterization was performed using 20 vegetation plots per site, where we measured: diameter at breast height (DBH), tree density (ind/ha, of trees with DBH > 10 cm), basal area, and tree height, as well as the density, DBH, cavity and tree heights of trees with cavities (considered trees with available cavities for secondary cavity-nesting birds).

We conducted a survey of cavity-nesting birds using point-counts in logged and unlogged sites during two breeding seasons: October to January of 2015–2016 and 2016–2017. For each breeding season, in two unlogged sites, we performed 30 point- counts (site 1 and 2), while at a third site we performed 26 point-counts. We used the same methods at three logged sites as well (Fig. 1). Similarly, at two logged sites, we conducted 30 point-counts (sites 2 and 3), while at the remaining site (site 1) we conducted 26 point counts (Fig. 1). We performed the point-counts every 150 m^49^, recording every cavity-nesting bird species seen or heard within a 50 m radius for 10 min between 07:00 and 11:00 am. Point-counts in consecutive seasons helped us to reduce possible observer bias in species detections and to average the values of both breeding periods^29,50^.

### Taxonomic and functional diversity analyses

With all point-counts, we calculated the richness and abundance of cavity-nesting birds for each treatment during the two years. We used the total number of species identified on the point-count to estimate species richness, and the maximum number of individuals detected as abundance (mean ± SE).

Based on Ibarra et al. (2017), functional traits associated with cavity-nesting birds in our analyses included categorical traits (nesting guild, trophic guild, and foraging substrate) and continuous traits (body mass and nest-tree size (DBH)). Categorical traits and body mass data were extracted from different publications^15,29,51,52^, while nest-tree size was obtained from our own data (Table 1). The nesting guild was divided into three categories: strong excavators (woodpeckers), weak excavators (trogons), and secondary-cavity nesters^29^. The trophic guild was divided into four categories: frugivore–insectivore, granivore, insectivore and omnivore^53,54^. Foraging substrate contained six categories: canopy, understory, ground, all forest strata, bark, and air^29,53^. Body masses were obtained from the Handbook of Birds of the World Alive^55^, and we averaged minimum and maximum values for each sex of each species. Traits in nest-tree size (DBH) were obtained in the field through nest searching performed at the same sites^28,52,56^.

**Table 1:** Trait values used to measure functional diversity parameters for avian cavity-nesting species from subtropical piedmont forests in Argentina.

English name	Scientific name	Nesting guild	Foraging guild	Foraging substrate	Nest-tree size (DBH, cm)	Body mass (mean g)
American Kestrel	Falco sparverius*	S	I	A	62.60	122.50
Black-banded Woodcreeper	*Dendrocolaptes picumnus*	S	I	X	57.32	82.50
Blue-crowned Trogon	*Trogon curucui*	WE	FI	C	59.75	51.00
Brown-crested Flycatcher	*Myiarchus tyrannulus*	S	I	X	35.51	29.00
Buff-browed Foliage-gleaner	*Syndactyla rufosuperciliata*	S	I	X	57.32	27.50
Cream-backed Woodpecker	*Campephilus leucopogon*	E	I	C	56.19	242.00
Dot-fronted Woodpecker	*Veniliornis frontalis*	E	I	U	42.34	35.00
Dusky-capped Flycatcher	*Myiarchus tuberculifer*	S	I	X	35.51	21.70
Golden-olive Woodpecker	Piculus rubiginosus**	E	I	C	46.78	59.50
Great Rufous Woodcreeper	*Xiphocolaptes major*	S	I	X	35.00	141.00
Green-cheeked Parakeet	*Pyrrhura molinae*	S	G	C	41.17	71.50
Narrow-billed Woodcreeper	*Lepidocolaptes angustirostris*	S	I	X	52.57	29.50
Olivaceous Woodcreeper	*Sittasomus griseicapillus*	S	I	X	57.32	14.00
Rufous Casiornis	*Casiornis rufus*	S	I	X	35.51	24.50
Scaly-headed Parrot	*Pionus maximiliani*	S	G	C	60.41	263.00
Streaked Flycatcher	*Myiodynastes maculatus*	S	I	C	53.88	43.50
Swainson’s Flycatcher	*Myiarchus swainsoni*	S	I	U	35.51	25.10
Toco Toucan	*Ramphastos toco*	S	O	C	68.28	680.00
Turquoise-fronted Parrot	*Amazona aestiva*	S	G	C	44.39	400.00
White-barred Piculet	*Picumnus cirratus*	S	I	U	44.95	9.15
White-eyed Parakeet	*Aratinga leucophthalma*	S	G	C	71.62	159.00
Yellow-collared Macaw	*Primolius auricollis*	S	G	C	52.54	245.00

Nesting guild: secondary-cavity nesters (S), weak cavity excavators (WE), strong primary excavator (E). Foraging guild: insectivore (I), frugivore–insectivore (FI), granivore (G), omnivore (O). Foraging substrate: air (A), bark (X), canopy (C), understory (U)^29,53^. ** Indicates species registered only in unlogged forest; * indicates those registered only in logged forest.

The functional diversity parameters included: Functional Richness (FRic), which represents the multivariate space filled by the functional traits in bird species present, regardless of the relative abundance of bird species; Functional Evenness (FEve), which represents the degree to which bird trait values are regularly distributed according to their abundance; and Functional Divergence (FDiv), which indicates the proximity of the most frequent functional trait values in the avian community. FRic and FDiv have no upper limits, whereas FEve varies between 0 and 1^38,57^. We also estimated the Community-Weighted mean (CWM), defined as the average of trait values in the community, weighted by the relative abundance of the species carrying each value, for body mass and nest-tree DBH^29,38,57^.

After standardization of traits, we estimated the functional diversity parameters for each treatment (logged and unlogged) in R (Version 1.0–11)^58^, using the *dbFD* function in package ‘FD’^59^. The taxonomic and functional diversity parameters were compared between treatments using an ANOVA and the Tukey-HSD posthoc test (F)^29^.

Finally, we compared our results with those obtained by Ibarra et al. (2017) for temperate forests of Canada and Chile with clearcutting (the authors also included partial logging) in order to compare different American forests. We reported the data obtained in the temperate forests of America along with our results, since the same analyses were carried out, with species richness, abundance, and functional diversity parameters (FRic, FEve, FDiv, CWM-biomass birds and CWM-nest tree size) obtained for both unlogged and logged sites.

## Results

### Site characteristics

In unlogged forests, we registered 54 tree species, and 22 of them had decay-formed cavities. In logged sites, we found 42 tree species, and 16 of them had decay-formed cavities. Averaged variable values (total trees/ha, basal area, DBH, and tree height) and dominant tree species are detailed in Fig. 1 and tables S1, S2, respectively.

### Avian taxonomic diversity

**Figure 2:**
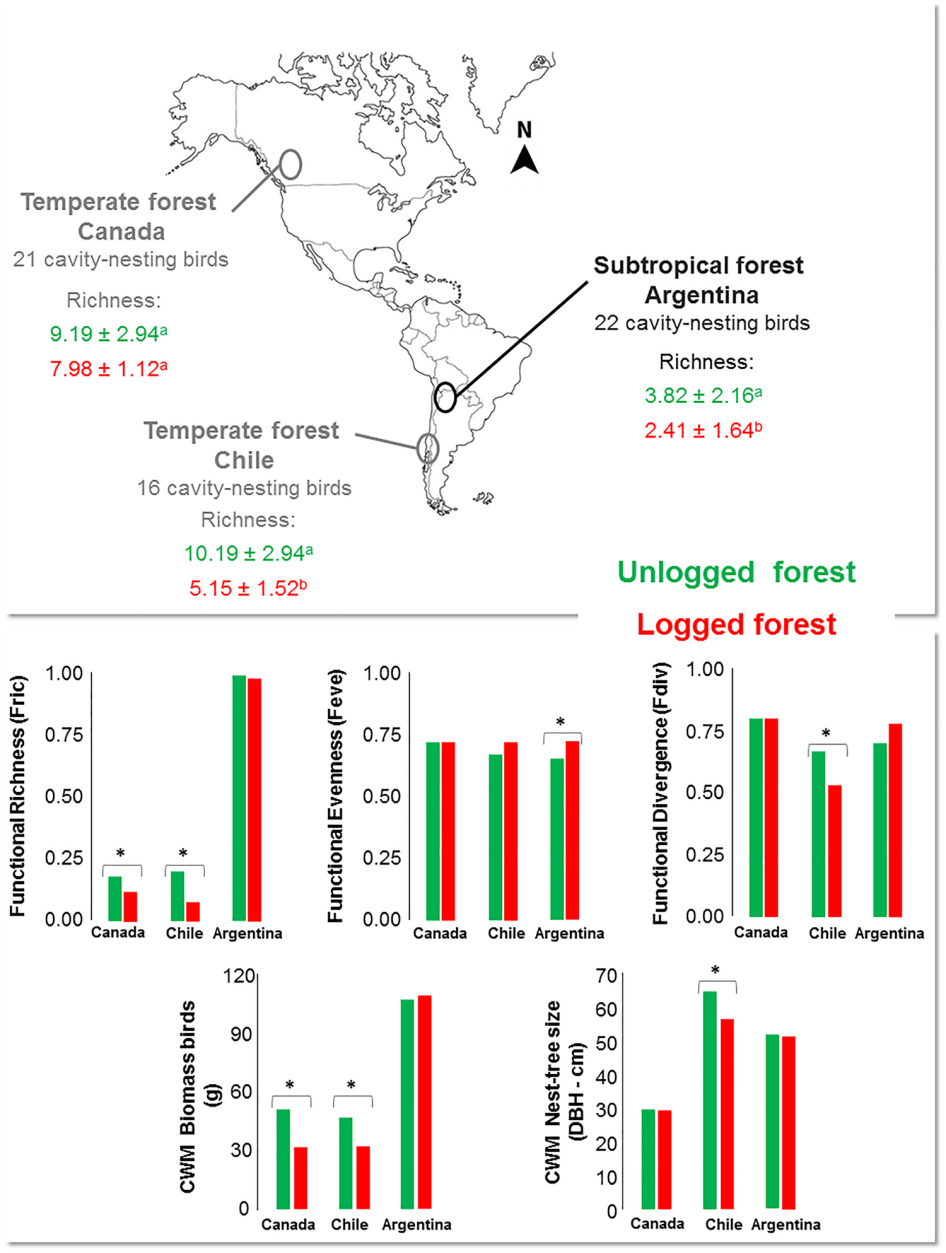
Functional richness and diversity in both temperate forests in Canada and Chile and subtropical forests in Argentina. The number of bird species and species richness for each site is also noted above: distinct letters indicate differences in the mean values between treatments. Functional diversity indices measured in all sites (below) and significant differences in mean values between treatments (*) are also depicted. This figure was produced using INFOSTAT software (//www.infostat.com.ar).

A total of 1673 individuals from 22 cavity-nesting bird species were recorded across the two breeding seasons. We registered 21 bird species at unlogged sites and 21 in ongoing logging sites. Species richness (F = 46.26; *p* < 0.001) and abundance (F = 64.55; *p* < 0.001) per point-count were significantly higher at unlogged sites (Fig. 2). Table 1 and S3 show details of cavity-nesting bird species registered and abundance in unlogged and logged sites.

### Functional diversity

We found no significant differences in functional richness (F = 4.50, *p* = 0.10) and functional divergence (F = 3.63, *p* = 0.13), but functional evenness was significantly higher in logging sites (F = 10.77; *p* = 0.03). The Community-Weighted Mean (CWM) showed no significant difference for body mass (F = 0.01; *p* = 0.93) and nest-tree size (F = 0.11; *p* = 0.75) (Fig. 2, and Table S4).

### Taxonomic and functional diversity in temperate and subtropical forests

Our results were partially similar to those reported for temperate forests (Fig. 2). In Canada, no significant differences were found in species richness between logged and unlogged sites (unlogged mean: 9.19 ± 2.94 vs. logged: 7.98 ± 1.12, *p* > 0.05), whereas Chile presented significant differences between treatments (unlogged mean: 10.19 ± 2.64 vs logged: 5.15 ± 1.52, *p* < 0.01) (Fig. 2). Functional richness values in temperate forests were close to 0, with significant differences between treatments (*p* < 0.01, Fig. 2). In subtropical forests, there were no significant differences between treatments and functional richness values close to 1 were observed (*p* = 0.10, Fig. 2). Functional evenness, on the other hand, was similar in temperate and subtropical forests (close to 0.75). Only in subtropical forests did we find significant differences regarding both treatments (logging sites presented higher values, *p* = 0.03). Similar values of functional divergence were also found in our three sites (close to 0.70–0.80), but treatments in Chile were different, being higher in unlogged forest sites (*p* < 0.05, Fig. 2). CWM-body mass turned out to be lower in temperate forests, with values close to 30 in logged and close to 50 in unlogged forests (*p* < 0.01, in Canada and Chile). Moreover, CWM-body mass in subtropical forests (value close to 110 g in unlogged and logged) presented no differences between treatments (*p* = 0.93, Fig. 2). CWM-nest tree size between treatments was significantly different only in temperate forests of Chile (*p* < 0.01, Fig. 2). In addition, this parameter was lower in Canada (close to 30 cm) compared to Chilean temperate forests and Argentinian subtropical forests, in which values exceeded 50 cm (Fig. 2 and Table S4).

## Discussion

Our results show how logging practices affect the richness and abundance of cavity-nesting birds, but maintain avian species composition (Fig. 2). Research conducted in other temperate and subtropical environments has reported a decrease in richness and abundance of birds in logged forests, mainly due to changes in forest structure (e.g. understory and canopy), food availability and suitable nesting sites^8,9,29,60^. This decrease may be explained by the low availability of nesting cavities^7^. Additionally, in these subtropical forests, logging has negatively affected the nest density of cavity-nesting birds^56^. This may also explain the decrease in the abundance of both excavators and secondary-cavity nesters, since logging reduces the availability of different and abundant trees, which may affect cavity-nesting ^10,27,47,52^.

We found that changes in species richness between treatments (unlogged vs logged) did not affect functional diversity. This indicates that changes in taxonomic richness caused by anthropogenic disturbances do not necessarily translate into changes in functional richness^31,34^. Thus, available ecological niches (e.g. trees available for nesting) are still being well exploited regardless of location^57^.

Avian cavity nesters in the studied forests have, therefore, similar ecological functions with equally defined ecological traits^34,61,62^. For example, in subtropical forests, FRic (unlogged: 0.99 vs. logged: 0.98, *p* = 0.10) was not affected by the number of species, since this index increased with species richness. Our FRic results differ from the ones reported for the temperate forests of Canada (unlogged: 0.18 vs. logged: 0.12, *p* < 0.01) and Chile (unlogged: 0.20 vs. logged: 0.08, *p* < 0.01), where this parameter was found to be significantly lower at subtropical logged sites, causing a decrease in the amount of functional niche volume filled by these bird species^29^. This supports the idea that in subtropical forests the functional richness observed in logging areas does not result in a decrease in the amount of functional niche volume occupied, and this niche volume is not colonized by generalist or opportunistic cavity-nesting birds^29^. It has been found that in disturbed forests, opportunistic species rapidly replace forest-specialized bird species, causing a decrease in functional traits^51,63^.

Higher Functional Evenness (FEve) values at subtropical logged sites (unlogged: 0.65 vs. logged: 0.72, *p* = 0.03) may be linked to habitat change and low tree availability, where birds are exploring new functional niches to reproduce and feed^38,57^. In temperate forests, no differences were revealed in FEve between logged and unlogged forests, also indicating that the functional niche volume filled by bird species can be relatively resistant to tree removal^29,38,51^. These high values of functional evenness in logged temperate and subtropical forest (≈ 0.70) are associated with more efficient resource use by cavity-nesting birds, indicating that abundance in traits drives ecosystem processes independent of taxonomic richness^64^.

Functional Divergence (FDiv) was not significantly different between treatments in subtropical forests (unlogged: 0.70vs. logged: 0.78, *p* = 0.13), indicating that subtropical forests lack birds with dominant functional traits (e.g. bird species that utilize the largest available trees for nesting in unlogged sites)^29,57^. The same results were obtained for Canadian temperate forests (≈ 0.80 in unlogged and logged), whilst in Chile, this parameter was significantly higher in unlogged sites (unlogged: 0.67 vs. logged: 0.53, *p* < 0.05) (Fig. 2). Thus, in temperate forests of South America, logging practices may cause differentiation of functional traits in bird species (e.g. highly values of DBH nest-tree used in unlogged forests) (Fig. 2). In subtropical and north temperate forests, however, logging may not reduce nest-tree availability and characteristic^38,40,57^.

The community weighted mean (CWM) for body mass presented no differences between treatments in subtropical forests, revealing that the species composition and body mass are maintained^3,38,57^. In contrast to our findings, the two temperate forests presented significant differences between treatments with lower bird body mass (≈30–50 g in temperate and ≈100–110 g in subtropical forests, Fig. 2). Despite the higher body mass of cavity nesters in subtropical forests, species were not excluded and this variable remained constant^29^, which may be explained by habitat type. Subtropical forests have a higher diversity of tree species available for nesting and foraging, thus, decreasing competition and maintaining bigger species^51,65^. CWM for nest-tree size used by birds in subtropical forests also appeared to be unaffected between treatments. This result possibly relates to bird usage of tall tree species for nesting (DBH > 50 cm, Fig. 2), despite the lower availability of trees^40^. Similar results were found in Canada, but not in Chile, where significant differences were reported. This may be due to in Chile, trees used for nesting had higher DBH values in unlogged sites, whereas in Canada, birds used trees of similar size in both treatments (Fig. 2 and Table S4). Besides this, in Canada, coniferous trees cannot form as many decayed cavities as deciduous trees, resulting in secondary-cavity nesters that depend mostly on cavities created by woodpeckers^15^. Secondary-cavity nesters in Chile, however, depend on large, decomposing trees, which are generally removed in logging sites^29^. Interestingly, in subtropical forests, these bird species use cavities generated by tree decay processes in different species of living trees^28,52^. This may allow birds to have more niches available for exploration, even in logged sites. This indicates that birds are using trees of similar size for nesting in both logged and unlogged sites in temperate forests of North America and subtropical forests of Argentina.

## Conclusion

Evidence from this study suggests that the ecological niches (e.g. nesting trees) are being well explored by birds in disturbed sites. This is an interesting finding since the analysis of richness and functional traits in trees available for secondary-cavity nesters (e.g. DBH, cavity height) showed lower values in logged sites^40^. Thus, despite the lower availability of tree species and the significant differences in the functional diversity of cavity trees, only trees with distinctive traits (such as DBH > 50 cm) were used in both sites, logged and unlogged. This is relevant for the management and conservation of logging sites in subtropical forests, which should consider the retention of large trees.

Finally, we highlight the importance of incorporating functional analyses under different forest disturbances, since changes in taxonomic richness do not always translate into changes in functional traits^31^. These analyses help in the clarification of the mechanisms and relationships between taxonomic richness and ecosystem functions^57^.

## Supplementary Information

Supplementary Information.
