# Supplementary material for: Responses of functional traits in cavity-nesting birds to logging in subtropical and temperate forests of the Americas
Source: Sci Rep. 2021 Dec 21;11:24309. doi: 10.1038/s41598-021-03756-0 (PMC8692622; doi:10.1038/s41598-021-03756-0)

**Responses of functional traits in cavity-nesting birds to logging in subtropical and temperate forests of the Americas**

Alejandro A. Schaaf, Daniela Gomez, Ever Tallei, Constanza G. Vivanco, Román A. Ruggera

**Table S1. Relative frequency of the main tree species at logged and unlogged sites in the Piedmont forests. Unlogged sites: 401.2 ± 79.32 tree/ha – Logged sites: 248.43 ± 65.04 tree/ha.**

|  |  |  |  |
| --- | --- | --- | --- |
| **Tree species** | **Frequency (%)** | | |
|  | **Unlogged forest** |  | **Logged forest** |
|  |  |  |  |
| *Phyllostylon rhamnoides* | 34.4 |  | 8.3 |
| *Calycophyllum multiflorum* | 11.6 |  | 3.4 |
| *Anadenanthera colubrina* | 28.5 |  | 22.4 |
| *Cedrela balansae* | 4.4 |  | 1.5 |
| *Myracrodruon urundeuva* | 4.3 |  | 1.9 |
| *Handroanthus impetiginosus* | 4.7 |  | 2.2 |
| *Ceiba chodatii* | 2.2 |  | 0.9 |
| *Myroxylon peruiferum* | 0.9 |  | 1.4 |
| *Amburana cearensis* | 0.4 |  | - |
| Snags | 0.8 |  | 0.5 |
| Other tree species | 7.8 |  | 57.5 |
| Total | 100% |  | 100% |

**Table S2. Relative frequency of the main tree species with potentially usable cavities for secondary-cavity nesting birds at logged and unlogged sites in the Piedmont forests.**

|  | | | |  |
| --- | --- | --- | --- | --- |
| **Cavity tree species** | **Frequency (%)** | | | |
|  | **Unlogged forest** |  | **Logged forest** | |
|  | **Total** |  | **Total** | |
| *Calycophyllum multiflorum* | **36** |  | **5** | |
| *Phyllostylon rhamnoides* | **18** |  | **12** | |
| *Myracrodruon urundeuva* | **8** |  | **8** | |
| *Anadenanthera colubrina* | **3** |  | **18** | |
| *Cedrela balansae* | **3** |  | **2** | |
| *Myroxylon peruiferum* | **2** |  | **-** | |
| *Amburana cearensis* | **2** |  | **-** | |
| Snags | **16** |  | **10** | |
| Other tree species | **12** |  | **45** | |
| Total | **100%** |  | **100%** | |

**Table S3. Cavity-nesting bird abundance (Mean ± SE) in unlogged and logged piedmont forests of northwestern Argentina. Significant differences among sites (Tukey-HSD posthoc test; *p*-value < 0.05) are shown in bold.**

|  |  |  |  |
| --- | --- | --- | --- |
| **English name** | Scientific name | **Abundance ± standar error** | |
|  |  | **Unlogged** | **Logged** |
| American Kestrel | *Falco sparverius* | **0.00** | **0.08 ± 0.01** |
| Black-banded Woodcreeper | *Dendrocolaptes picumnus* | **0.06 ± 0.02** | **0.03 ± 0.02** |
| Blue-crowned Trogon | *Trogon curucui* | **0.27 ± 0.55** | **0.13 ± 0.40** |
| Brown-crested Flycatcher | *Myiarchus tyrannulus* | 0.02 ± 0.01 | 0.02 ± 0.01 |
| Buff-browed Foliage-gleaner | *Syndactyla rufosuperciliata* | 0.30 ± 0.60 | 0.23 ± 0.47 |
| Cream-backed Woodpecker | *Campephilus leucopogon* | **0.18 ± 0.43** | **0.12 ± 0.39** |
| Dot-fronted Woodpecker | *Veniliornis frontalis* | **0.16 ± 0.03** | **0.05 ± 0.01** |
| Dusky-capped Flycatcher | *Myiarchus tuberculifer* | 0.03 ± 0.01 | 0.01 ± 0.01 |
| Golden-olive Woodpecker | *Piculus rubiginosus* | **0.02 ± 0.01** | **0.00** |
| Great Rufous Woodcreeper | *Xiphocolaptes major* | 0.06 ± 0.03 | 0.03 ± 0.01 |
| Green-cheeked Parakeet | *Pyrrhura molinae* | **1.00 ± 1.70** | **0.47 ± 1.15** |
| Narrow-billed Woodcreeper | *Lepidocolaptes angustirostris* | **0.48 ± 0.05** | **0.28 ± 0.04** |
| Olivaceous Woodcreeper | *Sittasomus griseicapillus* | **0.66 ± 0.05** | **0.43 ± 0.05** |
| Rufous Casiornis | *Casiornis rufus* | **0.06 ± 0.03** | **0.03 ± 0.01** |
| Scaly-headed Parrot | *Pionus maximiliani* | 0.22 ± 0.72 | 0.21 ± 0.62 |
| Streaked Flycatcher | *Myiodynastes maculatus* | **1.12 ± 1.21** | **0.33 ± 0.57** |
| Swainson’s Flycatcher | *Myiarchus swainsoni* | 0.21 ± 0.05 | 0.10 ± 0.03 |
| Toco Toucan | *Ramphastos toco* | **0.19 ± 0.55** | **0.13 ± 0.44** |
| Turquoise-fronted Parrot | *Amazona aestiva* | **0.33 ± 0.81** | **0.16 ± 0.64** |
| White-barred Piculet | *Picumnus cirratus* | 0.14 ± 0.39 | 0.16 ± 0.44 |
| White-eyed Parakeet | *Aratinga leucophthalma* | **0.53 ± 1.16** | **0.21 ± 0.91** |
| Yellow-collared Macaw | *Primolius auricollis* | **0.15 ± 0.62** | **0.06 ± 0.34** |

**Table S4. Functional diversity values for avian cavity-nesting species from temperate forests of Canada and Chile, and subtropical forest of Argentina.**


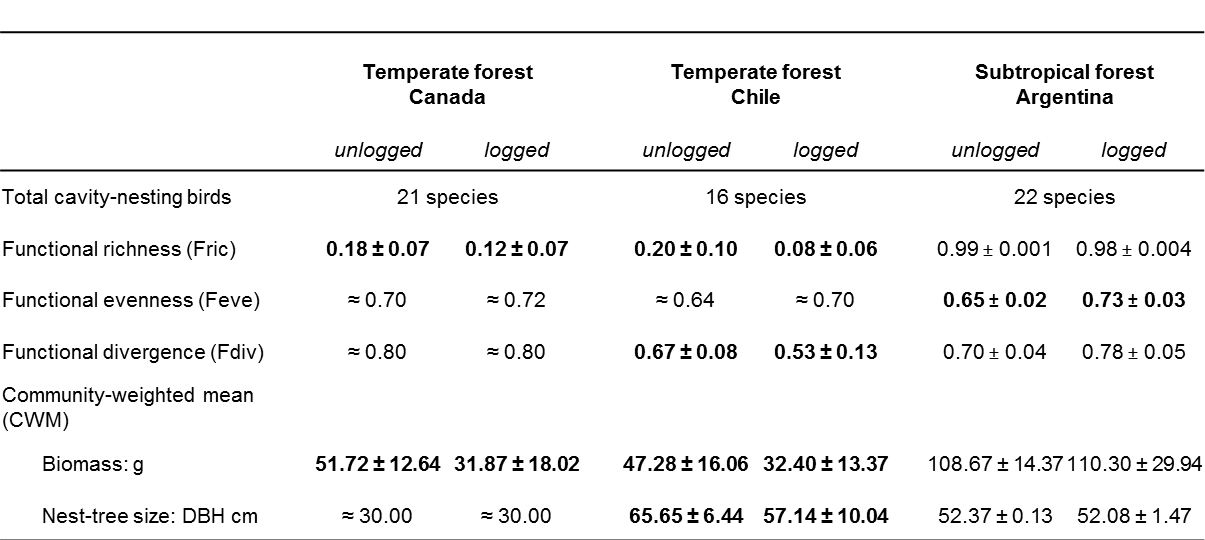

Supplement: Supplementary file 1 — Supplementary Information. [file 41598_2021_3756_MOESM1_ESM.docx]
